# Entrustable professional activities as a training and assessment framework in undergraduate medical education: A case study of a multi-institutional pilot

**DOI:** 10.1080/10872981.2023.2175405

**Published:** 2023-02-15

**Authors:** John A. Encandela, Lynn Shaull, Amy Jayas, Jonathan M. Amiel, David R. Brown, Vivian T. Obeso, Michael S. Ryan, Dorothy A. Andriole

**Affiliations:** a Associate Proessor of Psychiatry and Executive Director of the Teaching & Learning Center at the Yale School of Medicine, New Haven, Connecticut; b Senior Research Specialist at the Association of American Medical Colleges, Washington, DC, USA; c Research Analyst at the Association of American Medical Colleges, Washington, DC, USA; d Professor of Psychiatry and Senior Associate Dean at Columbia University Vagelos College of Physicians and Surgeons, New York, New York, USA; e Professor and Chief of Family and Community Medicine at Florida International University Herbert Wertheim College of Medicine, Miami, FL, USA; f Associate Professor of Medicine and Associate Dean for Curriculum and Medical Education at Florida International University Herbert Wertheim College of Medicine, Miami, FL, USA; g, Professor of Pediatrics and Associate Dean for Assessment, Evaluation, Research, Scholarly Innovation in Medical Education at the University of Virginia School of Medicine, Charlottesville, VA, USA; h Senior Director of Medical Research at the Association of American Medical Colleges, Washington, DC, USA

**Keywords:** Entrustable Professional Activities, Readiness for residency, Work-based assessments, Undergraduate medical education, Multi-institution pilot

## Abstract

In 2014, the Association of American Medical Colleges (AAMC) published 13 Core Entrustable Professional Activities (EPAs) that graduating students should be able to perform with indirect supervision when entering residency. A ten-school multi-year pilot was commissioned to test feasibility of implementing training and assessment of the AAMC’s 13 Core EPAs. In 2020–21, a case study was employed to describe pilot schools’ implementation experiences. Teams from nine of ten schools were interviewed to identify means and contexts of implementing EPAs and lessons learned. Audiotapes were transcribed then coded by investigators using conventional content analysis and a constant comparative method. Coded passages were organized in a database and analyzed for themes. Consensus among school teams regarding facilitators of EPA implementation included team commitment to piloting EPAs; agreement that: proximal EPA adoption with curriculum reform facilitates EPA implementation; EPAs ‘naturally fit’ in clerkships and provided opportunity for schools to reflect on and adjust curricula and assessments; and inter-school collaboration bolstered individual school progress. Schools did not make high-stakes decisions about student progress (e.g., promotion, graduation), yet EPA assessment results complemented other forms of assessment in providing students with robust formative feedback about their progress. Teams had varied perceptions of school capability to implement an EPA framework, influenced by various levels of dean involvement, willingness, and capability of schools to invest in data systems and provide other resources, strategic deployment of EPAs and assessments, and faculty buy-in. These factors affected varied pace of implementation. Teams agreed on the worthiness of piloting the Core EPAs, but substantial work is still needed to fully employ an EPA framework at the scale of entire classes of students with enough assessments per EPA and with required data validity/reliability. Recommendations stemming from findings may help inform further implementation efforts across other schools adopting or considering an EPA framework.

## Introduction

Entrustable professional activities (EPAs) are discrete observable assessable tasks that together attempt to define professional duties [1]. Introduced in graduate medical education (GME) in 2005, EPAs were intended to aid supervisors in making determinations about residents’ competence [2]. A resident is said to be ‘entrustable’ (or ‘ready to perform’ a given EPA with indirect supervision) incrementally, in relation to the complexity of tasks. Typically, residents become entrustable in relation to less complex tasks first, then in relation to more complex tasks throughout training. A final summative assessment would find a graduating resident ready to perform all tasks expected of an independently practicing physician without supervision; (or, in the case of residents moving on to specialty training, ready to take on this more complex level of education).

In undergraduate medical education (UME), the *graduating* medical student is, of course, not expected to perform the same tasks at the same level as a *graduating* resident. But like residents, medical students incrementally grow to be ready to perform various tasks with indirect supervision. The graduating medical student is deemed to be ‘ready to perform’ with indirect supervision the tasks expected of an intern – in other words, ‘entrustable’ at the level appropriate for an intern. A new trajectory of assessing incremental readiness then begins. For clarity, we use the phrase ‘readiness to perform as an intern’ rather than ‘entrustment’ as the preferred construct.

To define more clearly what ‘readiness to perform’ means at the level of beginning intern, the Association of American Medical Colleges (AAMC) published 13 Core EPAs for Entering Residency [3,4] (Table 1). To further support understanding and implementation of these EPAs, the AAMC published guides for faculty and learners [5] and curriculum developers [3]. Shortly after publication of these EPAs in 2014, the AAMC convened ten MD-granting U.S. schools (Table 2) [6] to determine feasibility of training and assessing medical students using the AAMC Core EPA framework. At each school, the deans and curriculum committee expressed a prerequisite commitment to pilot the Core EPA framework over multiple years and share information about processes and results.

**Table 1.** The 13 Core Entrustable Professional Activities (EPAs) for Entering Residency^3^.

EPA 1	Gather a history and perform a physical examination
EPA 2	Prioritize a differential diagnosis following a clinical encounter
EPA 3	Recommend and interpret common diagnostic and screening tests
EPA 4	Enter and discuss orders and prescriptions
EPA5	Document a clinical encounter in the patient record
EPA 6	Provide an oral presentation of a clinical encounter
EPA 7	Form clinical questions and retrieve evidence to advance patient care
EPA 8	Give or receive a patient handover to transition care responsibility
EPA 9	Collaborate as a member of an interprofessional team
EPA 10	Recognize a patient requiring urgent or emergent care and initiate evaluation and management
EPA 11	Obtain informed consent for tests and/or procedures
EPA 12	Perform general procedures of a physician
EPA 13	Identify system failures and contribute to a culture of safety and Improvement

**Note**: For extensive explanation of each EPA, please refer to the link in footnote 3. Also refer to the toolkits noted in footnote 8 for additional information.

**Table 2.** Pilot Schools.

Medical School	Affiliation	No. matriculants
Columbia University Vagelos College of Physicians & Surgeons	Private	138
Florida International University Herbert Wertheim College of Medicine	Public	135
McGovern Medical School at the University of Texas Health Sciences Center in Houston	Public	240
Michigan State University College of Human Medicine	Public	190
New York University Grossman School of Medicine	Private	108
Oregon Health & Science University	Public	140
University of Illinois College of Medicine	Public	315
Vanderbilt University School of Medicine	Private	95
Virginia Commonwealth University School of Medicine	Public	186
Yale School of Medicine	Private	104

Note: ^a^ *Source*: Association of American Medical Colleges. Table A-1: U.S. MD-granting medical school applications and matriculants by school, state of legal residence, and sex, 2021–2022. https://www.aamc.org/media/5976/download. Accessed January 3, 2022.

Selected schools represented a diverse range – from well established to newly created, small to large, rural and urban, and variably resourced medical schools. Variability meant that schools’ implementation teams would encounter some common and some unique challenges and successes. (Within the context of our eventual case study, reflecting on commonality and differences across multiple sites offered investigators the opportunity to understand dynamics between approaches and outcomes within and across institutions. This reflection also provided a window into understanding school readiness and feasibility for implementing Core EPAs.)

Hence, aims of the Core Entrustable Professional Activities for Entering Residency Pilot (hereafter, ‘Pilot’) were to: (1) test the feasibility of determining student readiness to perform Core EPAs under indirect supervision; and (2) share lessons learned with other institutions [6].

### The Pilot

Each school identified four individuals as primary members to lead local implementation and meet regularly with their multi-institution counterparts and AAMC supporting staff. Primary members typically included a clerkship director, a residency program director, an education leader in UME, and one other who could be a faculty member addressing faculty development, assessment, or another area related to EPA use. Schools expanded their *local* teams beyond these members as needed. Each team selected at least four EPAs to employ locally, making certain that, across all schools, each EPA was addressed by at least several schools. Some teams chose to implement more than four EPAs at their own discretion.

Each institution adjusted its program of assessment to incorporate EPA-specific measures at the curricular phases where they were most appropriate and feasible and could contribute to a determination of student readiness. Although this variance and variation in frequency of assessments occurred, some uniformity existed across schools. Pilot teams agreed to ‘introduce’ the concept of EPAs in pre-clerkship training, despite whether they had students practice EPAs and assess related skills during this period. The Pilot developed guiding principles [7], and one-page guidelines for each EPA [8], which helped all schools determine where in their curriculum the EPAs would be best situated and how the assessments would be developed. The EPAs themselves were not adjusted; those chosen by respective schools were employed exactly as written. Further, workplace-based assessments (WBAs)—assessments conducted by faculty observation of students in actual or simulated clinical settings – were the main source of student assessment data for all schools (though not the only source as discussed in our case study). All schools shared lessons learned at regular Pilot meetings and submitted semi-annual progress reports, which reviewed in aggregate, helped make clear the extent of uniformity and variance in implementation across schools.

### The case study

The purpose of this study was to describe the similarities and differences of implementation experiences across Pilot schools using a case study approach and guided by the research questions: how were Core EPAs put into operation at each school; what worked and did not work for schools individually and collectively; and, under what conditions and in what respects did aspects of pilot implementation work?

Although the focus of the study is on lessons learned about EPA implementation, implications for outcomes – such as Pilot schools’ ability to determine readiness of graduating students to perform all or a subset of the 13 EPAs under indirect supervision – is also a relevant consideration. A scoping review of studies on EPA use in UME suggests that the medical education community at large does not yet have adequate information about whether the 13 EPAs are the ‘definitive set’ with generalizability across UME [9]. It should be noted that the 13 Core EPAs were conceptualized as a preliminary set of UME EPAs that would be revised when empirical implementation data became available [4]. Our case study informs this ongoing discussion.

## Method

Qualitative case study methodology provided the basis for describing processes and effects of implementation across schools. A case study is used to ‘develop in-depth understanding of one or a small number of cases.’ [10] When multiple cases are included, focus is on common phenomena or activities across cases, comparing and contrasting experiences and developing a ‘unified understanding’ of progress [10]. In our study, we developed a case or ‘narrative’ of EPA implementation at each school, which we then compared and contrasted to form a unified understanding of successes, barriers, and lessons learned. This approach emphasizes the practical lessons that can be garnered from a current project or practice that can inform subsequent implementation of similar projects or practices. We used the *Program Evaluation Standards* [11] *and Critical Appraisal Questions for a Case Study* [12] to ensure that we met all research standards.

The study took place in 2020–21; AAMC Human Subjects Protection Program staff determined its exemption from further IRB review. To begin, two investigators with extensive training and experience in qualitative methods (LS, JE) analyzed written progress reports and Pilot meeting minutes generated over a seven-year period, documenting team updates to identify common processes and tools (or ‘mechanisms,’) that each school used to pilot EPAs. Conventional content analysis [13] was used by which these two investigators independently coded mechanisms with single words and phrases, then met to reach consensus until an exhaustive list surfaced. Mechanisms included, among others, activities to orient students to EPAs; faculty development for using EPAs in training and assessment; assessment tools; and data systems to support monitoring of student progress and related functions. These mechanisms provided a starting point for organizing interview questions for school implementation teams.

### Interviews

#### Participants

The four team members from each school were lead informants for interviews; others involved in local pilot implementation activities were invited to participate by primary team members. Each interview was conducted by some combination of the research team (always at least one non-AAMC and one AAMC staff member), all uniformly trained by the principal investigator (PI) (JE) in using a common interview protocol.

### Setting

An in-person interview was conducted with one school’s implementation team in February 2020. Soon after, intended in-person interviews were interrupted by the coronavirus-19 (COVID-19) pandemic. Consequently, implementation teams at eight additional schools participated in remote 2-hour+ WebEx© interviews between March and December 2020. Plans for the tenth school’s interview were interrupted several times by COVID-19 local case increases, which required team members’ full attention and ultimately precluded conducting an interview within the case study timeframe. However, analysis of this school’s progress reports identified the extent to which the team’s experiences aligned with interview findings, and the team lead was given the opportunity to review this manuscript. Interviews were audio recorded with participant consent, and recordings were transcribed by an outside service.

### Content

To facilitate interviews, our understanding of mechanisms was organized in an interview guide with related investigative questions. We asked teams to confirm the use of mechanisms, and when confirmed, further questions permitted informants to describe the context of Core EPA implementation and perceptions about successes, barriers, and lessons learned.

## Analysis

The initial interview transcript was open coded [13] by each of three investigators trained in qualitative methods (JE, LS, AJ), who then met to compare and reach consensus on codes. The same process was followed for remaining transcripts using a constant comparative method by which transcripts were incrementally re-reviewed and updated with agreed-on codes until a final coding scheme was developed [14]. Coded passages for each school were arranged in our textual database, using mechanisms for piloting EPAs as an organizing tool. The three investigators reached consensus on preliminary themes, which for ease of review were organized in two categories: *descriptive* (i.e., straightforward information about pilot implementation) and *phenomenological* (i.e., explanation of experiences in implementing the pilot). Themes developed for each school were provided to respective pilot team leaders as a member check [15], with changes made accordingly.

Meeting to review all school themes, investigators identified: (1) common themes across two or more schools; (2) competing themes representing different school experiences with common phenomena; and (3) unique themes for single schools. Thematic analysis ended when no new themes were identified, thus indicating data saturation [16]. Key findings were then derived from themes, and a final member check with school teams was conducted.

### Reflexivity

The authors are related to the Pilot: three from AAMC, four from school implementation teams, and one evaluation expert, the PI from one of the ten schools but not on its primary implementation team, and thus, not a member of the Pilot.

## Results

Findings fit into five categories: (1) administration; (2) curricular placement of EPAs, assessment, and entrustment; (3) data management and visualization; (4) coaching; and (5) multi-institution collaboration. Considerations in each of these categories directly contributed to schools’ implementation successes and challenges and clarified similarities and differences in Pilot schools’ experiences. Specific findings are described below; direct interviewee statements appear in quotation**s.**

### Administration

#### Buy-in

Extent of EPA integration with curricula was related to administrative buy-in. Although highest-level administrators supported their local pilots, ‘enthusiasm’ and level of resources varied, ranging from actively to minimally involved administrators. Teams with actively involved dean-level administrators responsible for medical education curriculum (e.g., deputy or associate deans for UME) perceived this to be ‘essential for uninterrupted implementation success.’ For instance, an implementation team member explained,

I think that one thing that was really helpful and that would not have happened any other way, was that our vice dean … was a member of the team of four and went to the national meetings. Though he wasn’t involved … day to day … I think that he felt compelled by the model, which meant that he could line up institutional support. He really believed in it and was willing to go to bat.

Having Deans of Medical Schools interested and frequently informed of EPA implementation progress was thought to be ‘a bonus’ for ensuring success.

### Curricular reform and integration with existing systems

Like many other U.S. schools, UME curricular reform occurred in some of the Pilot schools in recent years to better align with competency-based education [2]. Of the Pilot schools, two had reformed curricula five or more years prior to, and the majority within 5 years of Pilot start-up in fall 2014; while one newly established school had only graduated its first class several years earlier. Teams agreed that if a school was already making major curricular changes, it was easier to implement EPAs than it would be if trying to incorporate them in long-established curricula.

Most school teams found ways to integrate the EPA framework into local systems that used combinations of milestones, competencies, and/or competency-based objectives to steer curricula, yet the extent of integration varied across schools. Though each team was expected to employ at least four EPAs, teams typically cross-walked existing skill assessments of milestones or competencies with all 13 Core EPAs to identify which skills were and were not already being assessed and, when assessed, with what measures. This process afforded schools ‘a sort of quality improvement opportunity’ to identify and reflect on areas of training that might be enhanced with EPA language, skills, and assessments.

### Curricular placement of EPAs, assessment, and entrustment

#### Preclinical placement of EPAs

Most Pilot schools conceptually introduced and sometimes provided students with opportunities to become familiar with and practice EPAs in pre-clerkship activities. For example, Pilot school pre-clerkship faculty commonly taught and assessed students as they conducted a patient history and physical exam (EPA 1) with standardized patients, simulations, and case-based discussions. These opportunities were helpful in readying students for concentrated skill training and workplace-based assessments (WBAs) later in clerkships. Pre-clerkship introduction was viewed as an opportunity to ‘instill early-on in students the value of skill observation and feedback’ by faculty. In several cases, EPAs with limited opportunities to be practiced in clinical years were introduced preclinically as ‘aspirational’ to raise awareness and clarify future residency skill expectations (e.g., EPAs 10–13 in Table 1).

### Clinical placement of EPA assessments

Clerkships were perceived as ‘natural’ places to introduce EPAs. An implementation team member explained:

‘ … there did seem to be particular “homes” for some of the EPAs … for clerkships, taking history, doing physical exam [EPA 1], doing oral presentations [EPA 6], differential diagnosis [EPA 2]. This is a really core feature of clerkships and has been a core feature of clerkships even before EPAs.’

Though team members acknowledged the long-standing nature of focusing on these skills in clerkships, they believed the ‘value added’ by a concerted Pilot included: (1) ability to consider the skills addressed by the 13 Core EPAs, or a subset of these, to help students prepare for residency; (2) emphasis on direct, workplace-based observation of skills and just-in-time formative feedback, rather than waiting until mid or end of rotation for assessment; and (3) tracking student progress longitudinally with EPA-related data.

Two existing assessment scales were identified for use by Pilot members: the Modified Chen scale measures the level/amount of supervision the assessor believes would be needed the next time the student performs the EPA; and the Modified Ottawa scale measures the level of co-activity the assessor needed to render in actual performance of a skill by a trainee [8]. Though some variation across schools persisted, most settled on use of the modified Ottawa scale in response to faculty-assessor preference. Additionally, Pilot members developed one-page schematics in which expected learner competencies were delineated for each EPA [8]. Also included were examples of behaviors that would indicate either need for correction or evidence of competency achievement. Though not intended to be assessment instruments, teams found these schematics helpful in developing WBAs and ‘shared mental models’ among faculty for conducting assessments.

While not conceived as a probable outcome within the timeframe of the Pilot, it is important to note that no Pilot school depended *solely* on EPA-related WBAs to make determinations about student performance and progress in clerkships. WBAs for EPAs were used for formative assessment purposes and complemented assessment mechanisms already in place for some EPA-related skills (e.g., history-taking and physical examination); some schools decided to replace assessments that ‘were not working well’ with WBAs or other forms of skill assessment. Specific clerkships in which EPAs and WBAs were implemented varied (e.g., at some schools in all clerkships, at others in singular clerkships, etc.). Adoption depended on leadership and faculty buy-in and extent of opportunities for observation and feedback (which varied considerably on an EPA-specific basis across the 13 EPAs).

Post-clerkship clinical rotations and electives at some schools afforded opportunities for EPA training and assessment. Examples include transition-to-residency courses and sub-internships in which EPAs requiring ‘higher-level’ skills (e.g., EPA-4, regarding ordering prescriptions and EPA-8 regarding conducting of patient handovers) may be best addressed. In fact, one school whose clerkships incorporated EPAs, ‘placed the most emphasis’ on Core EPAs in post-clerkship clinical training.

### Assessors and adoption of EPAs

Assessors included front-line clinical faculty, residents, community physicians, and advanced practice providers. In some cases, pre-clerkship teachers used EPAs and WBAs to observe and assess students. Approaches to faculty/resident development varied by school, though concentrated instruction on the EPA framework and WBAs for all EPA assessors was perceived to be the most successful approach for promoting assessment quality. Teams agreed that local pilot-team development and delivery of faculty development in EPA assessment was the ideal approach, rather than depending on leaders of discrete curricular components to provide faculty development for their respective faculty. A centralized approach assured more uniform understanding of EPAs and assessment across medical school faculty.

Buy-in from front-line faculty assessors varied within and between schools depending on several related conditions: (1) levels of enthusiasm and persuasion that curriculum leaders exhibited for EPAs, affecting earlier or later adoption by others [17]; (2) faculty perceptions of ‘intrusion’ that assessments would have in conducting clinical and other teaching activities; and (3) faculty perspectives regarding relevance and usefulness of EPAs for training students relative to current approaches. School teams speculated that if WBAs were perceived by faculty to be more time-consuming than impactful, faculty might conduct these assessments in a ‘performative’ way in which they quickly ‘go through the steps’ of assessment with little informative value for students. This, in turn, could lessen students’ enthusiasm for asking assessors to complete WBAs since at many schools, students were responsible for requesting their supervisor to complete a WBA for a specific EPA).

### Utility and limitations of EPA assessment data

Members of implementation teams who were part of the Pilot from its inception said they realized early on that implementation of EPA assessments for summative decision-making would be more complex than first envisioned. It was unlikely that sufficient numbers of assessments per EPA would be amassed to generate good-quality data supporting high-stakes decisions. (Lomis et al., 2017:769 corroborates this perception.) [6] Therefore, for the remainder of the Pilot, teams determined that they would continue to test the feasibility and value of using EPAs and WBAs for possible *eventual* summative decision-making beyond the course of the Pilot. However, some implementation team members referred to a recent pilot-study of summative decision-making within the context of the overall Pilot to assert that, in accordance with this study’s results, members had moderate confidence in ‘mock’ summative entrustment decisions for several EPAs commonly practiced in clerkships (especially EPA 1 and 6; see Table 1) [18]. By the end of the Pilot, most schools ultimately convened such ‘mock’ summative decision-making about student readiness.

Although not used for actual summative decision-making within the scope of the Pilot, systematic evaluation of available student assessment data offered insights into the content and function of clinical curricula. This reflection permitted schools to make needed programmatic adjustments. An interviewee expressed:

… getting sufficient data and making entrustment decisions is much more effective as a program evaluation tool and for being clear about expectation setting. And I think it also helps identify students that are having challenges. But the final [determination of entrustment for each student] is still the most difficult part.

Additionally, it is important to stress the value they placed in the *formative assessment* opportunity that assessment of EPAs afforded. Narrative comments stemming from WBAs were said to be especially helpful in (1) adding concrete support for explaining to students the rationale for grades/ratings they received from other types of assessments; and (2) giving students timely, practical information about how they may progress to the next levels of skill-competency. One team member explained:

I think the students were very happy to be given the opportunity to understand what was being expected of them, and I think that framing EPAs as skills that they would need to progress … was helpful]. The whole project was a really profound opportunity to focus on readiness for next steps in training and to frame the whole UME training as a series of transitions for which we aspire to readiness.

Implementation teams reported that, in addition to students being less enthused to participate if they perceived WBAs to be merely performative, they expressed trepidation if they believed that these assessments might be determinative of advancement. (Perceptions of EPAs and related assessments from medical student leaders at Pilot schools were explored in a separate study, which was sometimes referenced in our case study interviews.) [19] This reaction highlighted the need for faculty to reassure students of the value of WBAs for providing formative feedback. Teams stressed that such feedback should be not only corrective, but should also highlight performance done well, ‘always includ[ing] suggestions on how students may enhance performance.’

### Data management and visualization

#### Data collection and storage

Processes for collecting EPA assessment data varied among schools but most followed a general process – after performing an EPA, a student retrieved a WBA form on a portable electronic device and handed it to a frontline observer who provided an *ad hoc* entrustment-level rating and narrative feedback; this information was automatically transported to a data storage system.

Schools created electronic WBA forms using a variety of methods: a homegrown system function, Qualtrics©, or Google Forms©. Several switched from paper to electronic forms during the Pilot, indicating this to be ‘a difficult [to execute] but necessary step.’ Most schools depended on use of cell phones to access WBA forms. At schools with the resources to do so, students were provided with iPad Minis©; team members reported that their students considered these devices to be ‘the right size’ to fit in whitecoat pockets while also affording ample screen size.

Data storage systems varied among schools, with a little over half using homegrown and the remaining using commercial systems (e.g., Microsoft Access©, Tableau©, Box©). For some, homegrown systems for managing and visualizing data offered efficiency and were helpful in giving technology staff flexibility to customize dashboards to local needs. Others said, however, that building an entirely homegrown system is resource intensive and these systems can be ‘clunky,’ adding that the solution might be to combine existing parts of pre-packaged resources with homegrown systems tailored to local needs.

### Data visualization

Most schools developed dashboards that ‘culled’ WBA and other assessment data for individual students, then displayed data in visually accessible ways. Several schools permitted students to supplement data with Individualized Learning Plans (ILPs) in dashboards, allowing coaches and students to review assessment data together, after which students could immediately update ILPs.

Two school teams reported being very pleased with their dashboards; the leader of one asserted:

… I would say our [data system] is … the best I’ve seen. And it’s because we didn’t try to fit it into any package. Two members of technology team come to the bi-monthly meetings every single time. And so, they’ve been incredibly instrumental in building the WBAs, the coaching platform, the dashboard, everything along the way.

For most implementation teams, however, culling data then putting them in a form that was visually accessible was a major barrier. One interviewee stated,

I would say one of the biggest challenges there is just, we still haven’t accomplished the data visualization that would put [EPA assessment] information in a usable fashion in front of the advisors [and their students] … That’s really the technical challenge of getting the right information to them in a way that they can use it easily.

Assembling hardcopy summary reports became necessary for some schools, requiring significant staff time. An implementation team member described,

For us to do the analysis that we want to look at a student snapshot, the data ha[ve] to be all pulled out oftentimes manually in multiple reports. And then [staff] have spent 400 plus hours to clean it, reorganize it, move it around so that we can do the analysis we want. The original vision was for [data visualization to be automatically generated] within the [electronic] system. And that functionality didn’t exist.

Another team agreed,

I think we didn’t really appreciate all the work that would go into [creating effective data visualization] and the resources that it was going to require, and we’re continuing to move in that direction. It’s just taking us longer to get there.

Additionally, schools had trouble including pre-clerkship assessment data because of differences in data collection and storage mechanisms between pre- and post-clerkship data. Given these challenges, some schools stopped using their dashboards entirely, or revamped dashboards as resources became available.

In summary, attempts to create data visualization approaches ‘did not work’ for most schools, and for those for which it did work, it was because schools invested resources in data systems with highly engaged and technically skilled data teams. In this sense, the Pilot afforded schools the opportunity to assess their visualization and technological capabilities that could have benefit for future programmatic decision-making.

### Coaching

Coaching models varied across schools, including coaches who: (a) formally focused on EPA progress; or (b) consistently incorporated discussions of EPA progress, along with discussions of wider-focused academic progression; or (c) addressed EPA progress in an ad hoc manner when coaches thought it would benefit students. A few schools provided general academic coaching with no formalized EPA coaching involved. Despite variation, school teams agreed that some level of coaching focusing on longitudinal academic progression is necessary for successful EPA adoption – a concept included in the Pilot’s guiding principles [7].

Teams acknowledged that ideally and if resources permit, there are two types—1) assessment coaches and 2) portfolio coaches – that would facilitate a ‘full-fledged’ EPA program. ‘Assessment coaches’ observe and give students just-in-time feedback in clinical learning settings. Primarily concentrating on formative feedback, they are typically ‘front-line’ teachers in clinical contexts. Also, these ‘Assessment coaches’ may be clerkship or course faculty who focus specifically on providing feedback on EPA skills but who are not (or are less) involved in ‘front-line’ clinical care. (A school using additional faculty as assessment coaches conveyed the drawback that these faculty often do not see students in direct patient interaction; instead, coaches depend on student reports of their WBA results and patient contacts to provide coaching.) Assessment coaches typically do not provide summative feedback over time, though their ratings and comments may be used by whomever provides such. Instead, ‘portfolio coaches’ offer longitudinal feedback. They typically do *not* see students they coach in clinical settings, but review WBA and other assessment data with students to provide guidance on student progress.

While various types and levels of coaching occurred across schools, only one school formally employed *and* paid both types of coaches, considered by other teams to be ‘the ideal.’ Variance in resources, availability of faculty, and school priorities all contributed to the extent to which coaching specifically addressed student progress towards readiness for the performance of a given EPA with indirect supervision.

### Multi-institution collaboration

The much-appreciated value of collaboration across Pilot schools was a theme that permeated all team interviews. Despite implementation barriers and in addition to successes, teams repeatedly noted the ‘irreplaceable value’ of having colleagues to rely on for feedback, brainstorming, and advancing their scholarly contributions. A member from one team explained:

I don’t think that we would have buy-in if one school was doing this on their own. The fact that this was ten schools moving forward together, even if they have distinct processes is what really helped this whole thing be successful.

A member of another team explained the benefit of collaborating on publications, noting that these publications will ‘help move the education community forward.'

Implementation team members noted some shared collaborative resources and guidelines, such as the earlier-discussed guiding principles [7] and one-page schematics [8] that provided a type of common ‘starting point.’ Yet, they also explained that variability of implementation across schools meant that teams ‘were on their own to figure out direction’ for elements of implementation for which there were no shared experiences. The team member offering this insight added:

… if we would have started with that focus, with more buy-in towards doing things systematically as much as possible, I feel like we would have moved the field forward a lot more quickly.

Teams agreed that if there had been resources allocated for schools to create and test a small set of *specific uniform* elements, such as the same assessment tools, faculty development materials, and coaching guidelines, collaboration might have had *even greater* yield.

## Discussion

Our case study elucidated some areas of consensus about facilitators of EPA implementation, as well as significant variation in schools’ pilot implementation experiences and results. To summarize, teams expressed consensus on: (1) the importance of a collaborative spirit by which all implementation teams committed to learning from local experiences and sharing lessons with one another, thus, bolstering individual schools’ accomplishments; (2) general recognition that the closer in time to major curriculum reform, the easier it may be for schools to implement an EPA framework; (3) a ‘natural fit’ for employing EPAs and WBAs in clerkships, though other potential places in the curriculum should be considered as well; and (4) the benefit of using Core EPAs to reflect on schools’ curricula and assessment systems in order to identify and fill gaps in both. Most teams agreed that EPAs provided the possibility (even if not fully realized) to offer relevant formative student feedback orally and in writing, more longitudinal relationships between students and faculty (especially coaches), and a common framework for providing guidance about what skills are needed for entering residency.

Another notable agreement among teams was that confidence in EPA-based assessment data to determine readiness for residency for whole classes of students has yet to be achieved. Schools were closer to having ability to determine readiness for some EPAs more than others. Conducting a patient history and physical exam (EPA 1) and providing an oral presentation (EPA 6) were assessed most frequently, producing more data per student. Larger amounts of data meant that determination of readiness for whole classes of students *in these skill areas* was a more achievable but still future goal. But for other EPAs, such as identifying system failures and contributing to a culture of safety (EPA 13), amounts of data were scant. Possible reasons were that faculty champions were less apt to adopt such an EPA in their curricular components and/or the clinical environments in which students functioned did not provide meaningful opportunities to perform these EPAs. By the end of the Pilot, teams generally agreed that EPAs 10 through 13 (see Table 1) likely would need to be revisited and perhaps revised to make them more appropriate for determining graduating student readiness for internship.

Though we have yet to learn if all 13 EPAs and their assessments are appropriate or generalizable, it is of note that one multi-institution collaborative – Education in Pediatrics Across the Continuum (EPAC)—has produced an important finding in this regard. EPAC is an AAMC-facilitated collaborative that has pilot-tested advancement of students through medical school based on student achievement of defined outcomes rather than time. This initiative has demonstrated feasibility of implementation and assessment of all but one (EPA 12) of the 13 Core EPAs for making decisions about student progress among a very small, highly selective group of students [20]. Initiatives such as EPAC may provide the ‘natural’ follow-up of the Core EPA Pilot, integrating lessons learned from our pilot with further tests on the use of EPA-based assessments for high-stakes decision-making in UME.

Our case study also highlighted varying experiences among schools in levels of: (1) active participation in local implementation by education decision-makers; (2) faculty willingness to adopt an EPA framework both within and across institutions; and (3) resource and strategic commitment to data management and visualization systems and EPA coaching. These factors affected pace of implementation, with greater success occurring when there were early EPA champions and ample resources. Similarly, a recent scoping review focusing on studies of EPA implementation in UME found a high level of variability in how EPAs and their related assessment are used across and within three national contexts: the U.S., Canada, and Switzerland [21]. Authors found that ‘[m]ethods for developing EPAs, implementing EPA-based clinical curricula in UME, and assessing EPAs varied and no clear standard has yet emerged for UME clinical rotations.’ While local adaptation and ability to make local decisions is essential, educators and learners both may benefit from a level of uniformity in national frameworks, such as agreement on what constitutes readiness at the UME level and recommended *minimum* technology requirements to assure proper data management and visualization.

Based on insights associated with these findings, we offer the following recommendations (also condensed in Table 3) for schools early in or considering adoption of EPAs:

***When planning for EPA adoption:***

- Consider conducting a ‘readiness for change’ assessment before adopting an EPA framework [22]. (We discuss this recommendation in more detail below.)
- Consider timing adoption with (or as part of) other concurrent major change initiatives when administrators, faculty, and students are most ‘primed’ for change.
- Calculate education leadership investment as a fundamental consideration before adopting an EPA framework.
- Consider what role EPAs will play, if any, in skill-training and assessment in relation to other existing frameworks, such as milestones.

**Table 3.** Summary of recommendations for schools adopting or considering adoption of an EPA framework.

Planning	Conduct ‘readiness for change’ assessments when considering EPA framework adoption, determining levels of ‘change commitment’ and ‘change efficacy’ present in one’s school.
	Take advantage of the synergy that might occur with introducing EPA adoption with other large change initiatives.
	Consider dean- and curriculum-committee-level investment in EPA framework development before undertaking EPA adoption.
Implementing	Consider a diffusion of innovation model of implementation, starting with faculty ‘early adopters.’
	Take advantage of the ‘natural fit’ of some EPAs with clerkship training and assessment, but attempt to address EPAs where they fit best across the whole medical school curriculum, emphasizing vertical curriculum integration and longitudinal student/faculty relationships.
	Place value in using EPA assessment for formative learning of students while building towards a workable, fair, and trustworthy system in which EPA assessment may be used to inform summative decisions as well.
	Share experiences and resources with other schools and agencies supporting an EPA framework.

***Once you have decided to implement an EPA framework:***

- Consider a diffusion of innovation model of implementation, starting with faculty who are ‘early adopters’ who share successes and potential barriers with ‘middle and late adopters.’ [17]
- Consider early adoption of EPAs in clerkships (e.g., EPAs 1, 2, 6 in Table 1) where there is a ‘natural fit’; yet do not depend on clerkships alone for introducing EPAs. Some EPAs can be introduced as aspirational in pre-clerkships and as ‘higher-level preparation’ in the post-clerkship period (e.g., EPAs 4, 8 in Table 1). Considering opportunities for addressing EPAs across the curriculum can reinforce vertical curricular integration and longitudinal relationships between students and faculty (especially those who coach students on EPA progress). Moreover, curriculum mapping may help identify optimal places within the curriculum to introduce, teach, and assess specific EPAs.
- Use EPA-related assessments for formative learning for all students before they are used to make higher-stake promotion decisions. Establishing a system for formative assessment, though intensive, likely has a more attainable timeline compared to establishing a workable, fair, and reliable system of summative decision-making, which is not feasible to implement and sustain without wide institutional buy-in.
- Place value in the importance of peer-institution support by sharing insights, encouragement, and materials developed to implement EPAs.

The first recommendation involving a ‘readiness for change’ assessment elicited deeper conversation within our case study team. We found that adequate *change commitment* [22] was likely present among pilot schools. Though dean-level leadership varied in amount of direct participation thus effecting pace of adoption, Pilot and local teams were ‘all in’ throughout the Pilot, committed to sharing emerging lessons. Change commitment was reinforced by a national call to improve preparedness of graduates for day one of residency to improve patient safety [23]. The pilot schools tackled this critical issue by meeting together in extensive discussions and championing local implementation.

Perceptions of *change efficacy*, [22] or perceived capability, varied more widely across schools. Determining such capability would require school leaders to take stock of task demands, resource perceptions, and situational factors related to a proposed shift in operations that would be required by EPA adoption. It was not possible to measure or even know the appropriate level of change efficacy required of schools at the start of the Pilot since UME adoption was innovative and largely undefined. Pilot teams ‘learned as they proceeded’ that full implementation would require substantial revisions to assessment and advisement programs, curricular structure, data visualization, and student advancement and promotion policies [6]. The ‘unknowns’ regarding what resources and strategies might be needed at the onset of the Pilot made it challenging for schools to completely assess their school’s capability for initiating and sustaining an EPA framework.

Teams described that despite their commitment to change, resource scarcity, competing school demands, and technological conundrums can negatively impact EPA implementation. On the other hand, some explained that resource availability and recent positive experience with change enhanced the possibility of EPA adoption. Willingness to innovate can have a positive *snowball effect*, fostering eagerness for future change. For instance, several Pilot schools were members of the American Medical Association’s Accelerating Change in Medical Education (ACE) [24], a consortium of schools that likely prepared participants for subsequent change projects (such as EPA adoption) since ACE engagement provided substantial funding and familiarity with the process of change.

At the local school level, a snowball effect was also observed. Faculty who adopted EPAs and WBAs earlier in pilot implementation had opportunities to influence future adopters – not so much around the need for later adopters to witness complete and undisputed success in using EPAs, but more with reference to witnessing curricular/assessment change without untoward consequences. Hence, we hypothesize that successful change leads to greater change efficacy, further reinforcing change commitment.

### Limitations

Our study has notable limitations. Interviews with school leaders, selected because of their intimate experience in implementing EPAs, likely did not represent perceptions of all school stakeholders. Though one interview included a student representative, we were primarily dependent on school teams to report their students’ perspectives as understood by these teams. During interviews, the Pilot was in its sixth year, introducing the possibility of recall bias. Additionally, though we suspect that participants were encouraged to be open about challenges, it is possible that some challenges were minimized due to perceived negative consequences in sharing this information with anyone outside their own institutions. We strived to offset these concerns with our assurance of confidentiality, promising not to identify individuals and not to describe any individual school approach in relation to specific findings without the respective school team’s permission. We offered participants the ability to submit information privately and confidentially to the PI. We organized interviews and began data collection with queries about implementation mechanisms known to be used by the teams rather than conducting completely open-ended interviews, and we shared the interview questions with teams prior to meeting with them so that they could refer to documentation to help with recall.

Another limitation involved members of the case study team who were participants in the Pilot, either as implementation team members or as AAMC staff supporting the Pilot. Closeness to the Pilot had the potential to introduce bias in deriving and interpreting findings, though it also provided deeper insight into the dynamics of the Pilot. The potential for bias was offset by securing a non-Pilot member as the PI with experience in evaluating multi-institutional initiatives, who introduced methods known to counterbalance bias. These approaches included PI-led training for case study members in qualitative data collection prior to interviews; independent investigator coding of transcripts prior to consensus sessions to establish final codes; facilitated reflexive conversations in the interpretation of data involving all case study team members; and several rounds of member checks by which we shared our interpretations of findings with implementation teams and adjusted/clarified findings based on their feedback to ensure greater trustworthiness of findings.

A scoping review of studies of EPA use in UME correctly points out that the AAMC’s 13 Core EPAs and Pilot ‘were developed for U.S. MD-granting medical schools; hence, findings from the Pilot may not generalize to medical education systems outside of the United States or to other programs (e.g., osteopathic programs).’ [9] This limitation holds true for our study’s findings. Fortunately, other medical education organizations are developing EPAs relative to their unique contexts [25,26]. Despite differences in national and training program contexts prompting the need for differing sets of EPAs and assessment approaches, there may be an emergent ‘generalizable’ lesson. Unrestrained variability in implementation and results across schools is likely without a concerted effort to provide centrally facilitated guidance and resources, which would give adopting schools an equal chance for success in determining students’ readiness for residency.

## Conclusion

School implementation teams provided us extensive information about their experiences with EPA implementation which we organized in findings in five categories: administration; curricular placement of EPAs, assessment and entrustment; data management and visualization; coaching; and multi-institution collaboration. In their accounts, team members shared having moments of hopefulness and challenge in interaction with the Pilot. They had lively discussions about the potential that determining entrustment had for ensuring a more prepared graduate that could mitigate against the need to quickly get new interns ‘up to speed’ upon arrival for residency. It also became clear as the pilot progressed that WBAs based on EPAs were not immune to the usual challenges with assessment. Difficulty in obtaining enough WBAs per EPA per student and persistent concerns about validity and reliability of entrustment decisions make it clear that there is still work to be done to *fully* implement a Core EPA framework across *whole classes* of students at the Pilot schools. To promote feasibility for a larger number, revisions to the EPA framework may be warranted with perhaps a smaller subset of EPAs (possibly integrated with other frameworks such as milestones) that are the most feasible to attain and relevant for residency readiness.

Beyond our set of recommendations for individual schools seeking to implement an EPA framework, our findings may have implications for other multi-institution change processes affecting medical education generally. Even when there may be agreement on frameworks and commitment to piloting large-scale change, there is likely to be local specificity, which can produce wide variability in extent and success of implementation, and outcomes of these efforts, such as that experienced by the Pilot. We recommend that schools involved in large-scale changes *collaboratively* develop specific tools and strategies, thereby increasing the possibility of achieving generalizability of results and shared understandings of adjustments that might need to be made. When selecting schools to participate in large pilot efforts and/or supporting these schools to implement and study change, organizing agents should consider both school commitment to adopt *and* individual school perceptions of feasibility and capacity to make the changes required. While some schools will have the needed capacity, there may be lower-capacity schools that will benefit from supplemental resources to optimize opportunities for success across all participating schools.
